# Large Eczema Area and Severity Index response at 2-year follow-up of patients treated with dupilumab in routine care of atopic dermatitis: results from the national registry TREATgermany

**DOI:** 10.1093/skinhd/vzaf075

**Published:** 2025-10-01

**Authors:** Luise Heinrich, Thomas Birkner, Susanne Abraham, Sascha Gerdes, Tatjana Honstein, Sigrid Müller, Matthias Augustin, Inken Harder, Thomas Schaefer, Andreas Pinter, Anne Bong, Jutta Ramaker-Brunke, Tilo Biedermann, Michael Sticherling, Hannah Gorriahn-Maiterth, Knut Schäkel, Beate Schwarz, Sven Quist, Mohammad Asefi, Nicole Adler, Maren Stahl, Michael Schulz-Kiesow, Florian Schenck, Thomas Werfel, Stephan Weidinger, Annice Heratizadeh, Jochen Schmitt

**Affiliations:** Center for Evidence-Based Healthcare, Faculty of Medicine and University Hospital Carl Gustav Carus, Technische Universität Dresden, Dresden, Germany; Center for Evidence-Based Healthcare, Faculty of Medicine and University Hospital Carl Gustav Carus, Technische Universität Dresden, Dresden, Germany; Center for Evidence-Based Healthcare, Faculty of Medicine and University Hospital Carl Gustav Carus, Technische Universität Dresden, Dresden, Germany; Department of Dermatology, University Allergy Center, Medical Faculty Carl Gustav Carus, Technische Universität Dresden, Dresden, Germany; Center for Inflammatory Skin Diseases, Department of Dermatology and Allergy, University Hospital Schleswig-Holstein, Campus Kiel, Kiel, Germany; Department of Dermatology and Allergy, Hannover Medical School, Hannover, Germany; Center for Evidence-Based Healthcare, Faculty of Medicine and University Hospital Carl Gustav Carus, Technische Universität Dresden, Dresden, Germany; Institute for Health Services Research in Dermatology Hamburg, University Medical Center Hamburg Eppendorf, Hamburg, Germany; Center for Inflammatory Skin Diseases, Department of Dermatology and Allergy, University Hospital Schleswig-Holstein, Campus Kiel, Kiel, Germany; Practice Dr med. Thomas Schaefer and Dr med. Doreen Belz, Derma Koeln, Cologne, Germany; Department of Dermatology, Venereology and Allergology, Clinical Research, University Hospital, Frankfurt am Main, Germany; Practice Dr med. Anne Bong, Emmerich, Germany; Practice ‘Die Hautärzte’ Braunschweig, Braunschweig, Germany; Department of Dermatology and Allergy, School of Medicine, Technical University of Munich, Munich, Germany; Department of Dermatology, University, German Center for Immunotherapy, Erlangen, Germany; Practice Dermasana, Karlsruhe, Germany; Department of Dermatology, University Hospital, Heidelberg, Germany; Practice Dr med. Beate Schwarz, Langenau, Germany; Dermatology Clinic, Helix Medical Excellence Center Mainz, Mainz, Germany; Dermatology Study Center Hunsrück, Simmern, Germany; Hautärzte am Fastnachtsbrunnen, FÄ Nicole Adler, Mainz, Germany; Practice Dr med. Maren Stahl, Osterode, Germany; Practice Dr med. Michael Schulz-Kiesow and Dr med. Inken Reimers, Lübeck, Germany; Dermatology Center, Hannover, Germany; Department of Dermatology and Allergy, Hannover Medical School, Hannover, Germany; Center for Inflammatory Skin Diseases, Department of Dermatology and Allergy, University Hospital Schleswig-Holstein, Campus Kiel, Kiel, Germany; Department of Dermatology and Allergy, Hannover Medical School, Hannover, Germany; Center for Evidence-Based Healthcare, Faculty of Medicine and University Hospital Carl Gustav Carus, Technische Universität Dresden, Dresden, Germany

## Abstract

The TREATgermany registry was set up in 2016. We provide results on long-term dupilumab therapy in adults with moderate and severe atopic dermatitis (AD) in a routine care setting. The analysis cohort of 294 patients is one of the largest cohorts with 2-year follow-up data on treatment effects of dupilumab obtained from an AD registry to date. We report the Harmonising Outcome Measures for Eczema (HOME) core outcome set. The Eczema Area Severity Index (EASI) 75 and EASI 90 response rates for patients on treatment at month 24 were 78.8% and 51.2%, respectively.

Dear Editor, The TREATgermany registry was set up in 2016 and is the largest atopic dermatitis (AD) registry in Europe. A total of 1849 patients [mean (SD) age 39.8 (14.8) years, 837 (45.3%) female] were enrolled by 65 recruitment sites between June 2016 and July 2023. Here, we provide results on long-term dupilumab therapy in adults with moderate and severe AD in a routine care setting, extending our previous reports on short-term follow-up^1^ and drug survival.^2^ Some results from observational studies on longer term treatment with dupilumab are available.^3–6^ Our analysis population is one of the largest cohorts with 2-year follow-up data on the treatment effects of dupilumab obtained from an AD registry to date.

We present results from 294 patients [mean (SD) age 40.9 (14.5) years, 124 (42.2%) female] who were initiated on dupilumab at or after enrollment in the registry and were followed for 2 years [regardless of whether the patient was continuously treated with dupilumab throughout those 2 years (*n* = 237) or therapy was discontinued (*n* = 57) within that period]. Patients who received additional systemic therapies at or following the dupilumab start visit were included. The outcomes presented include the Harmonising Outcome Measures for Eczema (HOME) core outcome set,^7^ and were scheduled to be assessed every 3–6 months. Outcomes obtained after discontinuation of dupilumab were excluded from the calculation of the summary statistics. A sensitivity analysis for the Eczema Area Severity Index (EASI) response rates was conducted, which included all patients and classified patients from the time of dupilumab discontinuation as nonresponders in order to assess a possible selection effect due to the use of an on-treatment cohort in the main analysis.

In total, 225 patients (76.5%) had received systemic therapy for AD before they were initiated on dupilumab. Systemic glucocorticoids (*n* = 180, 61.2%) and ciclosporin (*n* = 106, 36.1%) were most frequently used. Patients had a high disease activity at the therapy start visit with a mean EASI score of 21.0, a mean Patient-Oriented Eczema Measure (POEM) score of 19.4, a mean Dermatology Life Quality Index (DLQI) score of 13.3 and a mean Itch Numerical Rating Scale (NRS) in the past 3 days score of 6.4 (Table 1).

**Table 1:** Outcomes for the dupilumab cohort over 2 years of follow-up in the TREATgermany registry

Outcome	Dupilumab start visit	3 months ± 2 weeks	6 months ± 4 weeks	12 months ± 6 weeks	18 months ± 6 weeks	24 months ± 6 weeks
Number of patients under dupilumab therapy	294	189	180	179	164	165
EASI, mean (SD)	21.0 (13.2)	6.1 (7.2)	3.9 (3.9)	3.8 (4.5)	3.0 (3.2)	3.1 (4.6)
EASI ≤7	33 (11.3)	137 (72.5)	152 (84.4)	153 (85.5)	147 (90.7)	144 (88.3)
EASI 50		149 (80.1)	156 (87.6)	157 (89.2)	150 (93.8)	147 (91.9)
EASI 75		92 (49.5)	122 (68.5)	124 (70.5)	122 (76.3)	126 (78.8)
EASI 90		41 (22.0)	58 (32.6)	74 (42.0)	71 (44.4)	82 (51.2)
POEM, mean (SD)	19.4 (6.4)	8.5 (6.3)	7.2 (5.1)	7.1 (5.5)	6.6 (5.3)	6.2 (5.7)
DLQI, mean (SD)	13.3 (7.4)	5.1 (5.6)	3.9 (4.6)	3.9 (4.4)	3.5 (4.2)	3.5 (4.5)
NRS Itch in the past 3 days, mean (SD)	6.4 (2.5)	3.0 (2.3)	2.8 (2.2)	2.7 (2.1)	2.4 (1.9)	2.4 (2.1)
NRS Itch in the past 3 days ≤4	67 (22.9)	148 (78.7)	142 (79.3)	149 (85.1)	139 (86.9)	134 (83.2)
NRS Peak Itch over the last 24 h^a^ (*n*)	14	23	35	44	60	81
Mean (SD)	5.5 (3.4)	3.0 (2.7)	2.4 (2.6)	2.6 (2.4)	1.9 (2.2)	2.1 (2.2)
RECAP^a^ (*n*)	14	23	35	44	61	80
Mean (SD)	17.9 (7.1)	6.8 (5.5)	5.5 (5.9)	6.5 (5.4)	5.0 (4.8)	4.9 (5.1)
Number of patients (including those who had discontinued dupilumab)	294	190	187	195	183	197
EASI response sensitivity						
EASI 50		149 (79.7)	156 (84.3)	157 (81.8)	150 (83.8)	147 (76.6)
EASI 75		92 (49.2)	122 (65.9)	124 (64.6)	122 (68.2)	126 (65.6)
EASI 90		41 (21.9)	58 (31.4)	74 (38.5)	71 (39.7)	82 (42.7)

Data are presented as *n* (%) unless otherwise indicated. DLQI, Dermatology Life Quality Index; EASI, Eczema Area Severity Index; EASI 50, ≥ 50% improvement in EASI from baseline; EASI 75, ≥ 75% improvement in EASI from baseline; EASI 90, ≥ 90% improvement in EASI from baseline; NRS, numeric rating scale; POEM, Patient-Oriented Eczema Measure. ^a^The outcomes NRS Peak Itch over the last 24 h and RECap for AtoPic eczema (RECAP) were introduced later in the patient questionnaire, which explains the smaller sample sizes.

The mean EASI score decreased to 3.1 (85% improvement) at month 24, with 88.3% having an EASI score ≤7, indicating a clinically highly meaningful response in clinical signs. The mean DLQI score reduced to 3.5 (74% improvement) at month 24, providing evidence of improved quality of life. The mean POEM sum score, reflecting symptom frequency and severity, decreased to 6.2 (68% improvement), as did the NRS Itch in the past 3 days score with a mean of 2.4 (63% improvement, 83.2% with NRS Itch ≤4). Improvements could also be seen for NRS Peak Itch over the last 24 h and RECap for AtoPic eczema (RECAP).

The EASI 75 and EASI 90 response rates for patients on treatment at month 24 were 78.8% and 51.2%, respectively. Generally, the outcomes improved markedly by month 3 and continued to improve until a plateau was reached around month 6. Our results align with those of other observational studies referenced above, even though we included patients without an adequate washout phase (patients with a therapy switch, *n* = 39) and patients with concomitant therapy, *n* = 15 (i.e. short-term administration of ciclosporin or systemic glucocorticoids).

We performed a sensitivity analysis to establish a lower threshold for the EASI response rates under a most conservative assumption regarding the reason for discontinuation of dupilumab (i.e. assumed nonresponse). The resulting rates at month 24 (EASI 75 of 65.6% and EASI 90 of 42.7%) constitute somewhat smaller, but still substantial, measures of effectiveness of dupilumab. These results are remarkably similar to the results reported by Boesjes *et al.*^6^

Ocular problems were reported for 107 (36.4%) patients, whereby 88 patients (29.9%) had at least one record of conjunctivitis. The severity of conjunctivitis was mild for about one-third and moderate for the other two-thirds of patients. Dupilumab was discontinued due to conjunctivitis in 13 patients (4.4%).

The results demonstrate that dupilumab was a milestone innovation for the treatment of AD that substantially improves long-term treatment. Conjunctivitis is the most frequently reported adverse event. Clinical effectiveness over a 2-year period under routine care was found to exceed the short-term effects reported in monotherapy clinical trials.^8^ The decrease in disease burden persisted in general for the entire 2-year period.

## Data Availability

The data underlying this article will be shared on reasonable request to the corresponding author.
